# Monitoring Intact Viruses Using Aptamers

**DOI:** 10.3390/bios6030040

**Published:** 2016-08-04

**Authors:** Penmetcha K. R. Kumar

**Affiliations:** Biomedical Research Institute, National Institute of Advanced Industrial Science and Technology, 1-1-1 Higashi, Tsukuba City 305-8566, Ibaraki, Japan; pkr-kumar@aist.go.jp; Tel.: +81-29-861-6773

**Keywords:** virus, antibodies, glycans, aptamer, biosensor, and surface plasmon resonance

## Abstract

Viral diagnosis and surveillance are necessary steps in containing the spread of viral diseases, and they help in the deployment of appropriate therapeutic interventions. In the past, the commonly employed viral detection methods were either cell-culture or molecule-level assays. Most of these assays are laborious and expensive, require special facilities, and provide a slow diagnosis. To circumvent these limitations, biosensor-based approaches are becoming attractive, especially after the successful commercialization of glucose and other biosensors. In the present article, I have reviewed the current progress using the biosensor approach for detecting intact viruses. At the time of writing this review, three types of bioreceptor surfaces (antibody-, glycan-, and aptamer-based) have been explored on different sensing platforms for detecting intact viruses. Among these bioreceptors, aptamer-based sensors have been increasingly explored for detecting intact viruses using surface plasmon resonance (SPR) and other platforms. Special emphasis is placed on the aptamer-based SPR platform in the present review.

## 1. Introduction

For the past few decades, viral diagnosis has become a necessary practice in viral epidemiology and the primary requirement for the clinical management of viral diseases. There are several reasons for this, including the significant progress in the development of specific antiviral therapies, the development of new diagnostic tools as an alternative to viral culture-based methods, and the emergence of new zoonotic and opportunistic viral infections. Because of the progress and challenges on these fronts, viral diagnosis plays an important role in understanding the epidemics and in containment of disease by appropriate therapeutic interventions using specific antiviral drugs. Viral diagnosis is routinely performed using either direct or indirect methods. In the former case, clinical samples are evaluated directly to determine whether intact viruses or their components, such as proteins or nucleic acids, are present. Alternatively, in the latter case, clinical samples are subjected to cell culture; cells, eggs, or animals are infected to isolate the virus or for serological detection using antibodies against the viral antigens or immunogens induced by the viral infections. Historically, viral diagnosis opted for indirect serological methods, including the complement fixation test, the hemagglutination inhibition test, immunofluorescence, the enzyme linked immunosorbent assay, and the Western blot assay. Although these assays are useful for viral diagnosis, they are limited to clinical labs, are laborious and time consuming, and lack sensitivity, possibly leading to delays in identifying the infectious agent and the treatment. Moreover, the serological diagnostic methods are less suitable for identifying newly emerging viral diseases, such as the Zika virus, swine and bird flu, Nipah virus, and Chikungunya virus, owing to their non-specificity in identifying subtypes or closely related strains.

To address these issues, over the past two decades, molecular diagnosis based on nucleic acid amplification has become dominant in viral diagnostics, primarily owing to the development of the polymerase chain reaction (PCR) method [1]. PCR provides millions of copies of DNA molecules, with two-fold amplification per cycle, using DNA polymerase. The amplified PCR products can be analyzed using either gel-electrophoresis or colorimetric methods. For the amplification of viral RNAs, the RNA is converted to cDNA by reverse transcriptase and is followed by PCR; this combination is termed RT-PCR. Using these amplification technologies, rapid and sensitive diagnostic protocols have been established against the human immunodeficiency virus (HIV) [2], hepatitis B and C viruses [3], and cytomegalovirus (CMV) [4]. PCR or RT-PCR has now become a gold standard method for viral diagnosis, and improvements have been incorporated, resulting in the nested-PCR, real-time PCR, digital PCR ligase chain reaction, and loop-mediated isothermal amplification methods. Although these nucleic acid amplification methods are now routine and common in viral diagnosis, they have shortcomings, such as the complex process for sample preparation (isolation of nucleic acids), the long times, the high cost, the potential for false positives, and the requirement for well-equipped diagnostic labs and trained personnel. To overcome these limitations and better manage viral diagnosis, biosensor-based platforms for viral diagnosis are attractive and provide rapid, direct, cheap, sensitive, and reproducible results for identifying a specific virus. The current most popular biosensor is the glucose sensor, which has facilitated better management of diabetes for the past three decades. The current review is focused on the progress towards direct detection of intact viruses, with a special focus on aptamer-based biosensors.

## 2. Monitoring Intact Viruses Using an Antibody as a Bioreceptor

**Figure 1:**
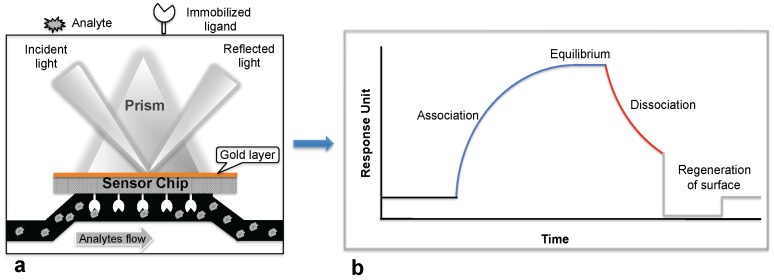
Surface plasmon resonance (SPR) biosensing platform: (**a**) SPR biosensing system; (**b**) Sensogram response observed upon the ligand interaction with the immobilized biomolecule.

Biosensor-based detection methods always utilize a specific bioreceptor surface to analyze either intact viruses or viral proteins. A common and widely explored bioreceptor surface has antibodies against the viral surface proteins or viral antigens. One of the earliest attempts to analyze an intact virus was reported by Schofield and Dimmock using a surface plasmon resonance (SPR) system [5]. The SPR system is an optical detection platform that uses prism coupling, and it allows characterization of the binding kinetics of biomolecular interactions in real time. To analyze the interaction between biomolecules, one interacting molecule is immobilized on the sensor surface (ligand), and its binding partner (analyte) is injected continuously into the buffer solution through the flow cell, resulting in analyte flowing over the ligand surface (Figure 1a). As a result of the analyte interaction with the ligand, the analyte accumulates on the surface and increases the refractive index. The change in refractive index is measured in real time, generating a plot of the response unit (RU) versus time (Figure 1b). The resulting responses obtained at different analyte concentrations are integrated to derive the rate constants (association, Ka; dissociation, Kd; and equilibrium dissociation, KD). The SPR system uses an optical method that analyzes refractive index changes at distances of approximately 300 nm from the surface. The first commercial SPR system was released to market in 1990. Since then, a number of SPR biosensor models have been released for analyzing various biomolecular interactions in a label-free environment. Among the biosensors platforms, the SPR platform was found to be more reliable, which allows us to measure biomolecular interactions with higher sensitivity and reproducibility.

**Figure 2:**
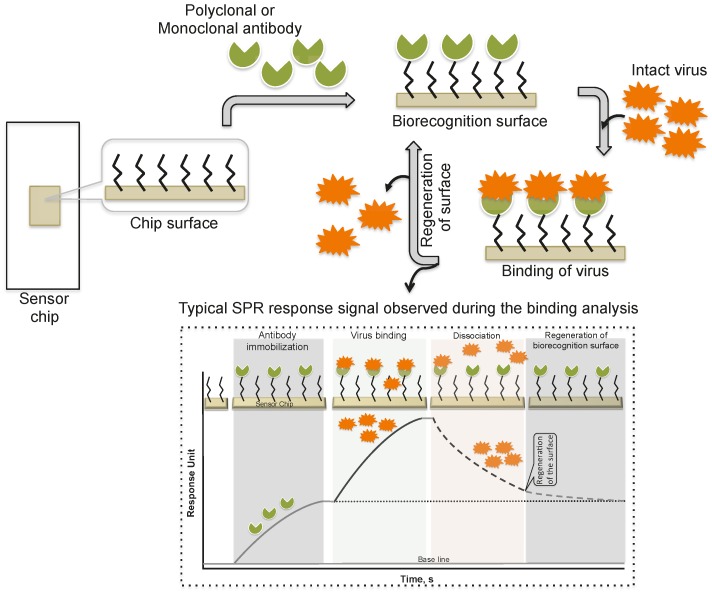
A schematic representation of analyses using antibodies as the biorecognition surface on the SPR platform.

Schofield and Dimmock [5] immobilized a monoclonal antibody via an amine-coupling reaction on the sensor chip (CM-5, which is coated with a carboxylated dextran polymer matrix). This monoclonal antibody (HC10) specifically recognizes the hemagglutinin (HA) derived from an influenza virus (A/fowl plague/Rostock/34 (H7N1)). The purified virus (A/fowl plague/Rostock/34 (H7N1)) was injected over the surface of the monoclonal antibody, and the response of the bound virus was observed (456 ± 21 RU). Interestingly, after the analysis, the monoclonal antibody surface could be regenerated by the injection of 0.1 M ammonium hydroxide solution, which stripped the bound virus. This procedure allows multiple analyses using the same monoclonal antibody surface without sacrificing the affinity towards the HA of A/fowl plague/Rostock/34 virus. The typical SPR analyses, steps, and output plot are summarized in Figure 2. Indeed, this work was the first demonstration that an entire viral particle with a size of approximately 120 nm could be analyzed through its interactions with monoclonal antibodies. Most viruses are smaller than the current limit for SPR measurements (approximately 300 nm); thus, it is possible that many plant and animal viruses can be analyzed. With these developments, for the past two decades, a few viruses have been detected using the protocol described above, the SPR platform [6,7,8,9,10,11,12], with some modifications, and these viruses are cataloged in Table 1.

**Figure 3:**
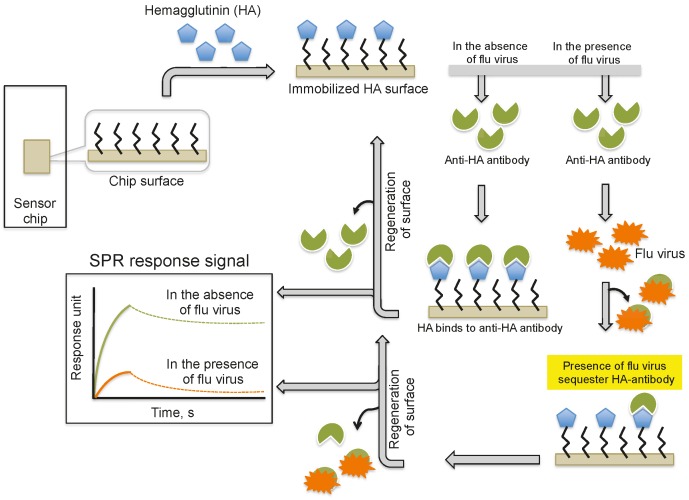
A schematic representation of analyses using antibodies as the biorecognition surface and inhibitors on the SPR platform.

Providing an alternative to the above analyses, Nilsson et al. (2010) reported a method for the quantification of influenza virus based on the inhibition of HA antibody binding (Figure 3). In this assay, HA derived from influenza viruses (A/H1N1, A/H3N2, and B) was immobilized on a CM5 chip using a standard amine-coupling protocol. Antibodies against the same strains of HA or antibodies that were mixed with the viral samples were injected over the immobilized HA surface. The response signal decreased with increasing viral concentration in the sample because the virus sequesters the antibodies and prevents binding to the immobilized HA. Using this strategy, the influenza virus detection limit was 0.5 μg/mL for all three viruses; thus, this method is approximately 10-fold more sensitive than the commonly used single radial immunodiffusion assay.

Similar to the above described strategies, the use of an antibody as a biorecognition surface has also been explored in other biosensor platforms for detecting intact viruses, including nanowire field effect transistor [13], interferometer [14], impedance-based [15,16,17,18], electrochemical [19], resonator [20], waveguide-mode [21] and surface acoustic wave [22] sensors (Table 2). Additionally, these biosensors showed promise for their application in the diagnosis of a wide range of viruses (300 nm), and they are suitable for typical viral sizes. However, the biosensors explored in the above studies used either polyclonal or monoclonal antibodies as the biorecognition method of binding to viral surface proteins. The sensitivity of these sensors depends entirely on the affinity and stability of the antibodies. Thus, it is difficult to compare the sensitivity and specificity of these sensors. Recently, many studies that relied on antibodies could not be reproduced owing to the cross-reactivity with other proteins, the variance between batches, or the instability of the antibody in the analysis conditions [23].

## 3. Monitoring Intact Viruses Using Glycan as Bioreceptor

**Figure 4:**
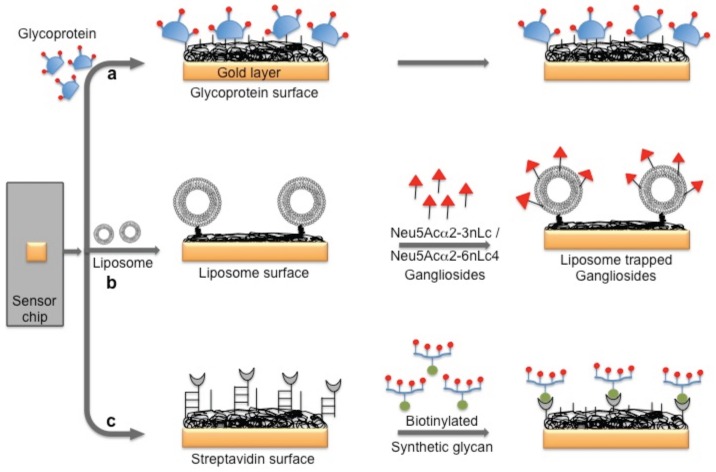
A schematic representation of the preparation of three possible glycan biorecognition surfaces on the SPR platform: (**a**) Glycoproteins as biorecognition surface; (**b**) Natural glycans as biorecognition surface; and (**c**) Synthetic glycans as biorecognition surface.

Several viruses carry glycoproteins on their surface to facilitate the specific recognition of glycans expressed on the host cell surface. Thus, in principle, to circumvent the problem of using an antibody for biorecognition on the surface, a glycan surface can be explored for virus detection, provided that its affinity and specificity for the viral surface proteins are higher than those of the antibody. In the past, three possible glycan surface methods have been attempted using the SPR platform to capture or detect viruses: (a) direct immobilization of glycoproteins that express specific glycan residues on their surface, which are recognized by the viruses; (b) immobilization of natural and purified glycans on the surface of liposomes (mimicking the natural surface); and (c) a multivalent synthetic glycan surface. In the case of reovirus analyses, a glycoprotein surface is being considered. The surface glycoprotein of reovirus is known to bind specifically to the α-linked sialic acid residues present on the host cell. For this analysis, the sialoglycoproteins expressed on the red blood cells (glycophorin and asialo-glycophorin) were used as a biorecognition surface in the SPR platform, and three strains of reovirus (T1L, T3C44, and T3C44-MA) that differ in sialic acid binding capacities were compared [24] (Figure 4a).

**Figure 5:**
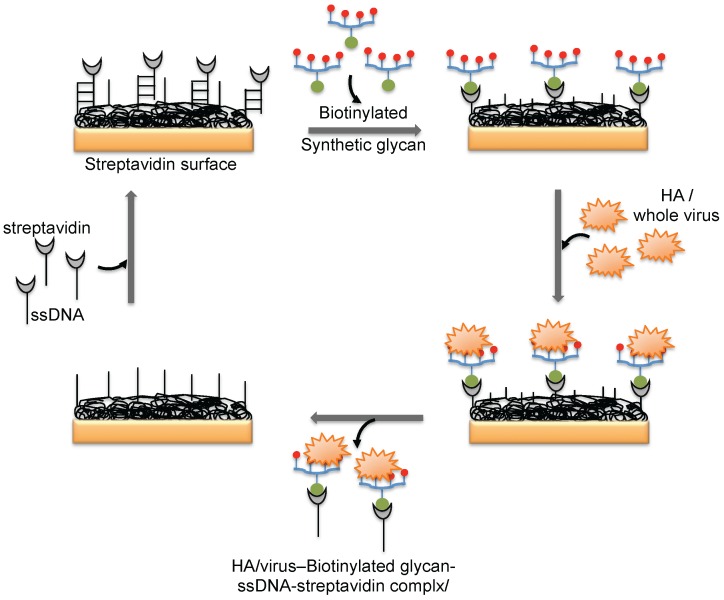
A schematic representation of the preparation of a glycan biorecognition surface for multiple analyses on the SPR platform.

The hemagglutinin (HA) protein of influenza virus binds specifically to the complex glycans on the host cell surface through a terminal sialic acid (Sia) with α2-3 and α2-6 linkages. Interestingly, the HA of avian influenza virus binds specifically to α2-3 Sia, which is preferentially expressed in the intestinal tracts of waterfowl. In contrast, human-adapted influenza virus binds specifically to α2-6 Sia, which is abundantly expressed in the epithelial cells of the human upper respiratory tract [25]. Human influenza virus (Human A/Aichi/2/6,8 (H3N2)) and avian influenza virus (Avian A/Duck/Hong Kong/313/4/78 (H5N3)) were evaluated for their glycan preferences using liposomes that incorporated gangliosides (Neu5Acα2-3nLc4 and Neu5Acα2-6nLc4) as the biorecognition surface in the SPR platform [26] (Figure 4b). Their analyses suggested remarkable differences in the binding kinetics of the two influenza viruses to the Neu5Acα2-3nLc4Cer and Neu5Acα2-6nLc4Cer gangliosides. For an alternative to the above two strategies, we previously explored synthetic glycans as a biorecognition surface for evaluating HA binding; the glycans were derived from either avian influenza viruses or human influenza viruses, and they could also be used for flu surveillance with the SPR platform [27,28,29] (Figure 4c). For screening to identify an appropriate glycan that can recognize HA with high efficiency, the three-dimensional structure of HA should be considered. The HA structure revealed that the HA is a homotrimer that possesses three glycan binding sites, which were estimated to be approximately 5 nm apart. One synthetic glycan tested in our study was a biotinylated tetravalent glycan that had four Sia glycan moieties at the distal end, and our building model suggested that the distance between the Sia glycans moieties was approximately 4 nm. Thus, the biotinylated tetravalent glycan would monovalently bind to HA but would capture other HA trimer using the three remaining Sia glycan moieties. For simplification of the analysis of the HA–glycan interactions, for multiple samples, and for a simple way to regenerate the biorecognition surface, we adopted a Biotin-CAP chip (Biacore), as shown in Figure 5. Using two synthetic glycan surfaces, (Neu5Acα2-3 Galβ1-4GlcNAcβ1-polyacrylamide (PAA)-biotin) and (Neu5Acα2-6 Galβ1-4GlcNAcβ1-PAA-biotin), HA derived from both avian and human influenza viruses were evaluated efficiently [27,28]. Recently, a glycan-based impedimetric biosensor was used to detect an influenza virus (H3N2). This biosensor was able to efficiently detect the viral particles (13 particles/μL) [30]. Although the glycan surface is an alternative to the antibody surface for biorecognition, the viral surface protein requires a glycan-binding site and must be recognized with high affinity and thus, the strategy is limited to few viruses, which meet such requirements.

## 4. Monitoring Intact Viruses Using an Aptamer as a Bioreceptor

Aptamers are known to bind with high affinity and specificity, and they are isolated from a library of nucleic acids by iterative rounds of selection and an amplification process known as the in vitro genetic selection strategy. Since the inception of the methodology more than two decades ago, several aptamers were selected against a wide range of targets, including simple ions, small molecules, peptides, proteins, organelles, and viruses [31,32,33,34]. The aptamer binding affinity and specificity that were achieved against the corresponding cognate targets were comparable or surpassed the affinity achieved between antibodies and antigens. Moreover, compared to antibodies, aptamers are smaller and easier to synthesize; additionally, several modifications can be incorporated, and they lack toxicity and immunogenicity. Because of these advantages, aptamers have been used for a number of applications, including imaging, diagnostic, and therapeutic purposes, which have been reviewed extensively [34,35,36,37]. Several high affinity and specific aptamers have been isolated against many viral proteins, including surface proteins of human pathogenic viruses [38,39]. Interestingly, some of these aptamers are able to distinguish very closely related families and subtypes [40,41,42,43,44,45,46,47]. Owing to the availability of high-affinity aptamers, it is possible to consider their application for the direct detection of intact viruses in virus-contaminated samples.

**Figure 6:**
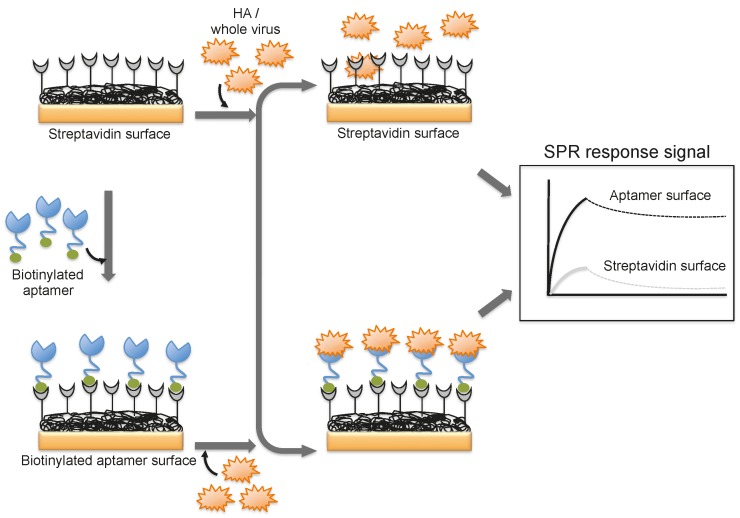
A schematic representation of analyses using an aptamer as the biorecognition surface on the SPR platform.

A DNA aptamer selected against the HA of avian influenza virus (A/Vietnam/1203/04) binds efficiently with an equilibrium dissociation constant of 4.6 nM [44]. Moreover, the aptamer showed specificity for binding to the HA derived from A/Vietnam/1203/04 and discriminated against all other HAs derived from other strains of H5N1 and also other subtypes of influenza A viruses [44]. The selected aptamer was then adopted for a biorecognition surface in SPR platform for the detection and evaluation of specific avian flu viruses (Figure 6). From the analyses, the concentration of avian influenza virus titers was quantitatively estimated to be in the range of 0.128–1.28 hemagglutination unit (HAU, is set as the minimum amount of HA or HA expressing virus required to cause agglutination of red blood cells and the titer of the virus solution, expressed as hemagglutination units per milliliters (HA Units/mL)), whereas the non-target influenza viruses (H1N1 (A/WileyLab/87), H2N2 (A/PA/chicken/1117-6/04), H5N2 (A/PA/chicken/85), H5N9 (A/WileyLab/85), H7N2 (A/PA/chicken/3779-2/97), H9N2 (A/WileyLab/87)) elicited an insignificant response signal [48]. Furthermore, poultry swab samples containing A/Vietnam/1203/04 virus titers were estimated efficiently, with a complete analysis in less than 1.5 h, which is shorter than the time for conventional methods of virus detection [48]. Although these studies suggest that whole viruses can be monitored by SPR, these approaches are limited to either a single or only a few samples because the sensor surface cannot be easily regenerated for the next round of analyses. To adopt the SPR platform for multiple sample analyses, it is important to employ suitable sequester reagents that allow simple regeneration procedures that restore the efficiency of the sensor without damaging the overall efficiency of the sensor chip.

**Figure 7:**
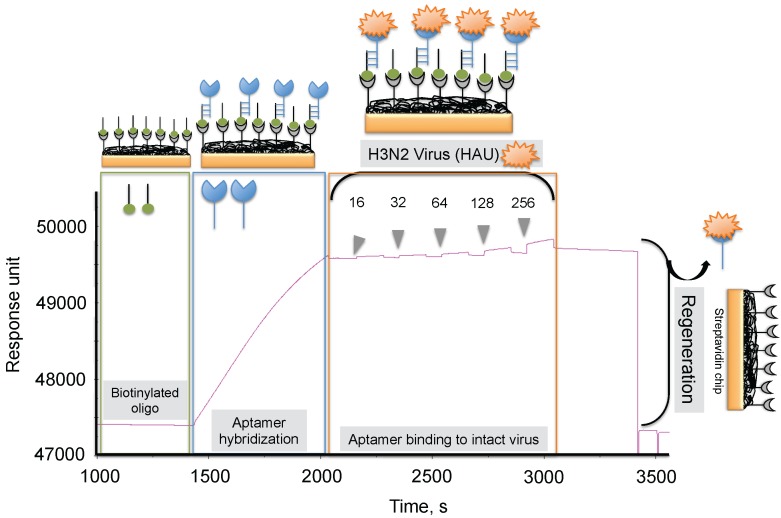
Typical response signals observed during the analyses of the aptamer-virus interaction on the SPR platform.

To progress in this direction, we reported an alternative methodology, which allowed us to regenerate the biorecognition surface of the sensor to analyze multiple samples. In this method, streptavidin (SA) was immobilized on the CM5 chip by an amine-coupling reaction followed by a biotinylated dT(24) oligo binding on the streptavidin surface of the chip. Our selected anti-H3N2 aptamer allowed hybridization to the oligo dT, which was extended by an A(24) residue tail at the 3’ end (schematic diagram shown in Figure 7) [41]. Over this surface, different amounts (16, 32, 64, 128, and 256 HAU) of A/Panama/2007/1999 (H3N2) virus were injected. A representative binding analysis of the entire cycle, including the immobilization of the aptamer, the virus binding analyses (single-cycle kinetics), and the regeneration step, is shown in Figure 7 [29]. All of the obtained response signals for different amounts (HAU) of virus were corrected by subtracting the responses of a control flow cell (where a complementary aptamer was immobilized) from the responses of an aptamer-containing flow cell. The response observed in the control flow cell was significantly lower than the aptamer cell responses during the above analyses. The observed response signals were plotted against each HAU for the influenza virus, showing a linear response with increasing HAU. The same chip was repeatedly used for >90 cycles. We believe that the response signal in the above studies could be improved further by considering a shorter length of the dextran matrix or other self-assembled monolayers (SAM). To test whether the response signal improved for a shorter length of dextran or SAM, we used a CM3 chip (which has approximately one-third of the thickness of the CM5 Biacore chip) and also prepared different SAMs (approximately 10 mm thickness) on a gold surface chip [29]. Using these chips, we repeated the analyses after immobilizing the SA, biotinylated oligo, and the aptamer, (as described above) with different amounts (HAU) of influenza virus. Our analyses found that both signal and the sensitivity improved on the chips when dextrans or SAM with a shorter chain length were employed [29]. Taken together, our analyses suggest that SAMs with a shorter chain length are preferable for analyzing intact viruses, even in the case of influenza virus, which is approximately 120 nm.

In another scenario, a DNA aptamer against an isolated influenza virus H1N1 (A/PR/8/34) [49] was linked covalently to a conductive polymer for the functionalization of microelectrodes in the microfluidic channel [50]. Upon virus binding to the DNA aptamer, the electrical signal changed at the electrode surface. The dynamic range of the sensor for detecting the influenza virus (H1N1) was approximately 10–10^6^ pfu/mL. The sensor not only detected the intact virus in clinically relevant samples (saliva) but also had a broad dynamic range, and the analyses were performed in approximately 15 min [50]. Thus, the described sensor has the potential to become a point-of-care (POC) device and could be readily adopted for the detection of other viruses using specific aptamers isolated against those viruses.

**Figure 8:**
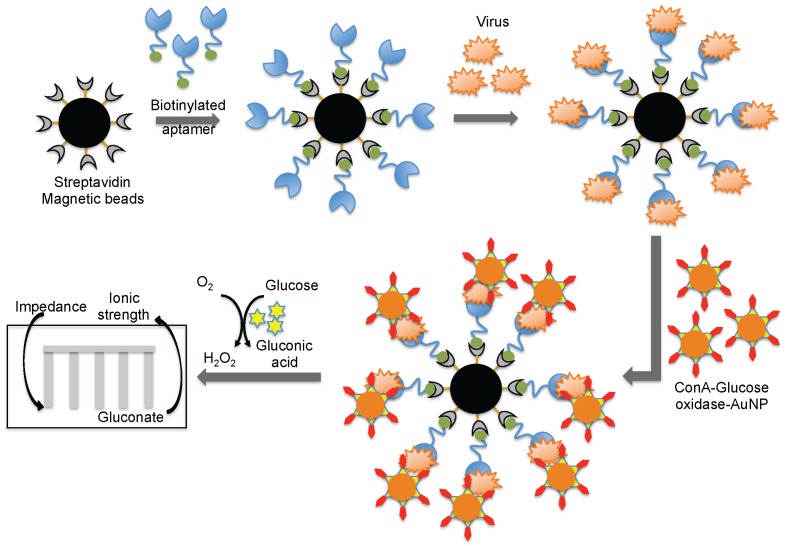
A schematic representation of analyses using an aptamer as the biorecognition surface on a different platform.

Wang et al. [44] combined both aptamer and glycan surfaces in an impedance biosensor for the direct detection of an intact virus. In this technique, a specific aptamer against the avian influenza virus (H5N1) that was developed previously [44] was captured on streptavidin-coated magnetic beads. When an avian influenza virus was present in the test sample, the aptamer was captured on the magnetic bead. Once the influenza was captured, a complex containing concanavalin A (ConA)–glucose oxidase immobilized on the 20 nm gold nanoparticle bound to the virus through the glycans of concanavalin A. The entire captured complex (aptamer–virus–ConA–glucose oxidase–gold particle) was transferred to an aqueous glucose solution to activate an enzymatic reaction to yield gluconic acid. The production of gluconic acid increased the ionic strength of the solution, which in turn decreased the impedance on a screen-printed interdigitated array electrode (Figure 8) [51]. Compared to biosensors using either antibodies or aptamers alone, the above described biosensor displayed better sensitivity (8 × 10^−4^ HAU/200 μL) [51]. A quartz crystal microbalance (QCM) based on the aptamer was also reported using the same aptamer that specifically binds to the avian influenza virus [52]. The QCM-based aptamer sensor detection limit for influenza was 0.0128 (HAU), and the analyses required approximately 30 min.

## 5. Conclusions

Timely surveillance of viral infections is important not only for predicting both endemic and pandemic threats but also for monitoring the evolution of viruses. Currently, viruses are being poorly monitored in many countries, and only a fraction of cultivated birds and animals are being subjected to surveillance, particularly for flu viruses [53]. Commonly used detection and surveillance methods include antigenic, serological, and agglutination assays. However, these assays exhibit low sensitivity and require large amounts of samples. Among the biosensing platforms for various biomolecular interaction analyses, surface plasmon resonance-based (SPR) sensing technologies are attractive because of their higher sensitivity, closed system for analyses, and the ability to use a label-free environment for analyses. Previously, antibodies have been explored as biorecognition surfaces for detecting intact viruses using the SPR platform. These studies have shown that intact viruses can be analyzed efficiently and rapidly. However, in the wake of instability problems associated with antibodies and other issues, alternative biorecognition molecules, such as aptamers, have been increasingly developed against a wide range of viral proteins [38,39] and have been evaluated in viral diagnosis applications [54,55]. Aptamers have a versatile nature, both in terms of adaptability to a wide range of biosensor platforms and suitability for multiple cycles of direct analyses of intact viruses. Although current studies have focused on flu viruses, the progress that has been made so far on this front may stimulate aptamer-based detection of other viruses and the development of additional biosensor platforms. Compared to other biosensors, the intact virus detection system must adopt a closed system (from sample to analyses) because of the infectivity of the samples. In this respect, the SPR platform, when combined with an aptamer as a biorecognition surface, appears to be the best choice because it allows sensitive detection of viruses in a label-free and closed environment. The original SPR system and the chips are expensive; however, inexpensive, small, and portable SPR systems are being developed [56,57] and are now available commercially [58,59,60]. Moreover, a number of efficient aptamers have been isolated against surface proteins of different viruses (Table 3) and undoubtedly their applications will be actualized in the future towards the development of biosensor for detecting intact viruses. Nevertheless, the incorporation of these recent developments into different platforms might soon lead to an aptamer-based biosensor that detects intact viruses.

## Figures and Tables

**Table 1:** Analyses of intact viruses using antibodies as the biorecognition surface on the SPR platform.

Virus	Approximate Size (nm)	Sensor Chip	Buffer	Bioreceptor	Reference
A/fowl plague/Rostock/34 (H7N1)	120	CM5	PBS	Monoclonal antibody	[5]
A/Puerto Rico/8/34 (H1N1)	120	SA	HBS-EP	Monoclonal antibody	[6]
Tobacco mosaic virus	180	Custom	Carbonate	Polyclonal antibody	[7]
Autograph californica multiple nuclear polyhedrosis virus	240	Custom	PBS	Monoclonal antibody	[8]
B/Brisbane/3/2007	120	CM5	HBS-EP+	Polyclonal antibody	[9]
A/Solomon Islands/3/2006 (H1N1)	120	CM5	HBS-EP+	Polyclonal antibody	[9]
A/PR/8/34 (H1N1)	120	CM5	HBS-EP+	Polyclonal antibody	[9]
A/Wsiconsin/67/2005 (H3N2)	120	CM5	HBS-EP+	Polyclonal antibody	[9]
A/PR/8/34 (H1N1)	120	Custom	PBS	Monoclonal antibody	[10]
Human cytomegalovirus	230	Custom	PBS	Monoclonal antibody	[10]
Human cytomegalovirus	230	CM3/CM5	PBS + 0.05% Tween20	Monoclonal antibody	[11]
Feline calicivirus (F-9 strain, VR-782)	30	CM3	HBS-EP+	Polyclonal antibody	[12]

**Table 2:** Analyses of intact viruses using antibodies as the biorecognition surface on different biosensor platforms.

Virus	Detection Method	Bioreceptor	Reference
Influenza virus A	Nanowire field effect transistors	Monoclonal Antibody	[13]
Herpes simplex virus-1	Interferometer sensor	Monoclonal Antibody	[14]
Rabies virus	Impedance spectroscopy	Polyclonal Antibody	[15]
Avian Influneza virus [A/Scotland 59 (H5N1)]	Microelectrode based Impedance spectroscopy	Polyclonal Antibody	[16]
Bacteriophages T7/ MS2	Nanowire electrochemical	Monoclonal Antibody	[17]
Avian Influneza virus [A/ck/PA/87 (H5N2)]	Impedance spectroscopy	Monoclonal Antibody	[18]
Bean pod mottle virus	Photonic microring resonators	Monoclonal Antibody	[19]
Avian Influneza virus [A/Scotland 59 (H5N1)]	Impedance biosensor	Monoclonal Antibody	[20]
A/Udon/307/1972 (H3N2)	Waveguide-mode sensor	Monoclonal Antibody	[21]
A/Brisbane/10/2007 (H3N2)	Waveguide-mode sensor	Monoclonal Antibody	[22]
Ebola virus (Zaire)	Surface acoustic wave	Monoclonal Antibody	[23]

**Table 3:** Aptamers isolated against the surface proteins of different viruses.

Virus	Apamer Target	Reference
Chikungunya/Dengue/West Nile	Viral Envelop proteins	[ 61]
HBV	Surface antigen	[62]
HCV	E2 Glycoprotein	[63]
Human cytomegalovirus	Whole virus	[64]
HIV	Gp120	[65]
HIV	Nucleocapsid	[66]
HSV-1	gD	[47]
HSV-2	gD	[67]
**Influenza A (H1N1)**		
California/2007/1999	HA	[45]
PR/8/34	HA	[24,68]
Brisbane/59/07	HA	[69]
California/04/09	HA	[69]
Singapore/6/86	HA	[69]
Georgia/20/06	HA	[69]
Perth/265/09	HA	[70]
**Influenza A (H2N2)**		
Japan/57	HA	[24]
**Influenza A (H3N2)**		
Panama/2007/1999	HA	[40,41]
Brisbane/10/07	HA	[69]
Wisconsin/67/05	HA	[69]
Moscow/10/99	HA	[69]
Texas/77	HA	[24,70]
Port Chalmers/1/73	HA	[24]
Guizhou/54/89	HA	[25]
**Influenza A (H5N1)**		
Vietnam/1203/2004	HA	[71]
Vietam/1194/2004	HA	[46]
Indonesia/05/2005	HA	[46]
Anhui/1/05	HA	[69]
**Influenza A (H7N7)**		
Netherlands/219/2003	HA	[46]
**Influenza A (H9N2)**		
Beijing/1/01	HA	[72]
Hebei/3/98	HA	[72]
**Influenza B**		
Johannesburg/05/99	HA	[42]
Tokio/53/99	HA	[73]
Jilin/20/03	HA	[73]
Rabies Virus	Whole virus	[74]
Rous sarcoma virus	Whole virus	[75]
Vaccinia	Whole virus/HA	[76,77]
	Surface protein	[78]
**Other viruses**		
Apple stem pitting virus	Coat protein	[79]
Alpha mosaic virus	Coat protein	[80]
Bacteriophage R17	Coat protein	[81]
